# The impact of family intimacy on college students’ negative emotions—the chain mediating effect of social support and psychological resilience

**DOI:** 10.3389/fpsyg.2026.1783366

**Published:** 2026-05-04

**Authors:** Dongmei Chen, Yanyu Zhu, Ziwei Shang, Wu Chen

**Affiliations:** 1 School of Marxism, Wuhan University, Wuhan, China; 2 Institute of Development and Educational Psychology, Wuhan University, Wuhan, China; 3 School of Arts, Wuhan University, Wuhan, China

**Keywords:** college students, family intimacy, negative emotions, psychological resilience, social support

## Abstract

**Introduction:**

Family intimacy—a core facet of family functioning—has been posited to protect college students from negative emotions, yet the mechanisms remain insufficiently specified. This study tested whether social support and psychological resilience independently and sequentially mediate the association between family intimacy and negative emotions.

**Methods:**

A cross-sectional survey was administered to college students across 33 provincial-level regions in China (3,589 questionnaires distributed; 3,010 valid; response rate = 83.87%; 37.9% male; age 19–29, M = 20.16, SD = 2.22). Measures included the Family Environment Scale—Chinese Version (family intimacy factor), DASS-21 (negative emotions), the Social Support dimension of the SRHMS, and the CD-RISC-10 (resilience). Internal consistency was acceptable (*α* = 0.955, 0.955, 0.900, and 0.949, respectively). Common method bias was assessed using Harman’s single-factor test (first factor = 31.23%). Zero-order correlations were computed and a chained mediation model was estimated with the PROCESS macro (Model 6), controlling for age and gender.

**Results:**

All key variables were correlated in the expected directions. Family intimacy negatively predicted negative emotions (*β* = −0.051, *t* = −2.919, *p* < 0.01) and positively predicted social support (*β* = 0.187, *p* < 0.001) and resilience (*β* = 0.043, *p* < 0.05). Social support negatively predicted negative emotions (*β* = −0.143, *p* < 0.001) and positively predicted resilience (*β* = 0.399, *p* < 0.001); resilience negatively predicted negative emotions (*β* = −0.269, *p* < 0.001). The total effect of family intimacy on negative emotions was −0.143 (95% CI [−0.190, −0.097]). The direct effect was −0.066 (95% CI [−0.111, −0.022]; 46.2% of the total). The total indirect effect was −0.077 (53.8%), comprising three significant pathways: via social support (−0.027, 95% CI [−0.037, −0.018]), via resilience (−0.012, 95% CI [−0.022, −0.002]), and the chain “family intimacy → social support → resilience → negative emotions” (−0.020, 95% CI [−0.026, −0.015]).

**Discussion:**

Family intimacy is associated with fewer negative emotions among college students both directly and indirectly through enhanced social support and resilience. These findings underscore the value of family-focused and campus-based strategies that strengthen family communication and support systems to cultivate resilience and mitigate negative emotional states.

## 1 Introduction

Adolescence and early adulthood represent pivotal stages in an individual’s development, marked by significant psychological and emotional changes. During this period, university students face a range of academic, social, and personal challenges that can contribute to increased levels of stress and negative emotions. Negative emotions such as anxiety, depression, and stress are common psychological responses among students navigating the transition to higher education. According to the China National Mental Health Development Report (2021–2022), college students, particularly in the 18–24 age group, have a significantly higher risk of developing mental health issues compared to other age groups (Chen and Li, 2021). This has sparked growing concern over how to effectively support students’ mental wellbeing during their university years.

Among the factors influencing mental health in university students, family dynamics, particularly family intimacy, play a crucial role in shaping emotional wellbeing. Family intimacy, characterized by emotional closeness, supportive interactions, and healthy communication patterns, has been shown to be a protective factor in promoting psychological resilience. Students from families with high intimacy levels are more likely to experience secure emotional support, which buffers against the negative impacts of academic and personal stress (Sharma and Tiwari, 2020). In contrast, those from less intimate family environments may struggle with emotional regulation and resilience when faced with life’s challenges, potentially leading to higher levels of negative emotions (Masten and Reed, 2002). Research indicates that supportive family relationships can promote emotional stability and help students develop better coping mechanisms (Beck et al., 1996).

Negative emotions in university students are often exacerbated by academic pressures. The rigorous demands of academic performance, combined with social and familial expectations, can create a high-stress environment. Previous studies have demonstrated that academic stress is one of the primary predictors of negative emotional states such as anxiety and depression in students (González and Hernández, 2017). However, the impact of family intimacy on this relationship remains underexplored. Given the increasing prevalence of mental health issues among college students, it is vital to understand how family intimacy influences the emotional experiences of students, especially as they cope with academic stressors. Social support and psychological resilience have been identified as key mediators in this relationship. Social support, both emotional and practical, is widely recognized as an essential resource in managing stress and improving mental health outcomes (Cohen and Wills, 1985). Family support, in particular, provides a foundation for students to develop positive coping strategies, reducing the likelihood of negative emotional experiences (Gerber and Pühse, 2015). Psychological resilience, the ability to adapt to adversity and recover from setbacks, is similarly influenced by family dynamics. Students with higher resilience are better equipped to manage stress, and family intimacy fosters the development of this resilience by creating a secure emotional environment (Schwarzer and Knoll, 2007).

The concept of family intimacy as a determinant of emotional wellbeing has been explored in various studies, yet few have focused on its role in university students’ negative emotions. Furthermore, although research supports the importance of social support and psychological resilience in buffering stress, the specific mediating roles of these factors between family intimacy and emotional outcomes remain unclear (Fletcher and Sarkar, 2013). Understanding these mechanisms is crucial for developing targeted interventions to improve mental health among students. Family intimacy, social support, and psychological resilience are all interconnected resources that can buffer against the harmful effects of stress and negative emotions, but the exact nature of their interplay needs further exploration (Li and Wei, 2018).

This study aims to fill this gap by investigating the relationship between family intimacy and negative emotions in university students. Additionally, it explores the mediating roles of social support and psychological resilience. It is hypothesized that family intimacy not only directly impacts students’ emotional wellbeing but also influences it indirectly through these two key psychological resources. The findings from this research have the potential to inform interventions that enhance family communication and emotional support, ultimately promoting mental health and resilience in university students.

## 2 Literature review and research hypotheses

### 2.1 The influence of family intimacy on negative emotions

Family is the initial context of an individual’s socialization, and parent–child interaction patterns have profound and lasting effects on physical and mental development (Fei et al., 1991). Family intimacy refers to the degree of emotional bonding among family members—reflected in emotional support, communication quality, engagement in shared activities, and mutual dependence (Thomée et al., 2007). As a core indicator of family functioning, higher family intimacy provides college students with an emotional secure base that is internalized into positive cognitive schemas, interpersonal skills, and coping resources, thereby promoting personality development and values formation (Guo et al., 2024). Students from highly intimate families typically receive greater emotional support, communicate more effectively with parents, and participate more deeply in family activities; this protective emotional climate fosters social competencies such as communication, collaboration, listening, and empathy (Yan and Wang, 2024). Empirical work further shows that family intimacy/adaptability predicts lower adolescent depression, whereas family conflict is linked to anxiety (Han and Geng, 2021). Thus, H1: Family intimacy is negatively associated with college students’ negative emotions.

### 2.2 The mediating effect of social support

Social support denotes the material and emotional resources individuals access through social networks (family, friends, colleagues, community) (Achenbach, 1966), which strengthen adaptation and buffer stress to promote mental health (Barlow and Durand, 2015). From a social–cognitive perspective, high-quality support fosters more positive appraisals so stressors are viewed as challenges rather than threats, thereby lowering anxiety and related negative affect (Li et al., 2005). Support networks also provide instrumental help and emotional comfort—forming a dual line of defense that sustains wellbeing (Finkelhor and Ormrod, 2001). Importantly, access to support is shaped by family relationship quality: family intimacy captures the emotional bonds and interactional quality among members (Olson, 2000; Yu et al., 2021) and is associated with fewer negative emotional states in student populations (Liu and Sun, 2010). Highly intimate families tend to feature open communication, responsive affect, and mutual trust, creating a safe, stable context that facilitates perceived understanding, acceptance, and help-seeking (Liu et al., 2014)—patterns observed across student cohorts (El-Gilany et al., 2019). In short, intimacy is not support per se but a precursor that elevates perceived and received support, enhancing emotion regulation and reducing negative emotions. H2: Social support mediates the effect of family intimacy on college students’ negative emotions.

### 2.3 The mediating effect of psychological resilience

Psychological resilience is a dynamic capacity to maintain functioning and adapt well under adversity by flexibly adjusting cognition, emotion, and behavior; it develops through person–environment transactions and is strongly shaped by supportive contexts (Elgar and McKinnon, 2015). Among college students, resilience predicts better coping with academic pressure and healthier appraisals and strategies, with broad benefits for wellbeing (Cuijpers et al., 2016). Meta-analytic evidence shows robust negative associations between resilience and depressive symptoms, and resilience-building improves emotional states and quality of life (Cuijpers et al., 2016), including via peer-based approaches (Gray and Sisto, 2025). Family intimacy—via tighter bonds and constructive communication—provides enduring security and belonging that support adaptive emotion regulation and positive appraisals (Hammen, 2009) Through emotional responsiveness, guidance, and positive feedback, intimate families strengthen self-efficacy and problem-solving beliefs, while modeling adaptive strategies that students internalize as psychological resources. Accordingly, H3: Psychological resilience mediates the relationship between family intimacy and college students’ negative emotions.

### 2.4 The chain mediating effect of social support and psychological resilience

Social support and resilience are tightly interlinked. Support from family, school, peers, and community is a key external resource for the development of resilience in youth (McLeod, 1997) and enhances coping confidence to buffer stress-related negative affect (Moffitt et al., 2008). Longitudinal and comparative studies in student samples show that higher support predicts greater resilience and more stable psychological states under life stressors (Wang, 2010; Song et al., 2014), with converging evidence from sleep/health and campus mental-health research (Schlarb et al., 2017; Xiao and Yang, 1987) and identity-wellbeing trajectories (Schwartz and Zamboanga, 2008). Educational perspectives likewise emphasize cultivating supportive climates to reduce negative affect (Yu, 2024), while deficits in support relate to maladaptive compensatory behaviors and poorer wellbeing (Weidman et al., 2012). In university settings, resilience and support jointly predict adaptation to stress and guide intervention design (Ju et al., 2013). Therefore, H4: Social support and psychological resilience jointly form a chain-mediating pathway linking family intimacy to reduced negative emotions in college students.

**Figure 1:**
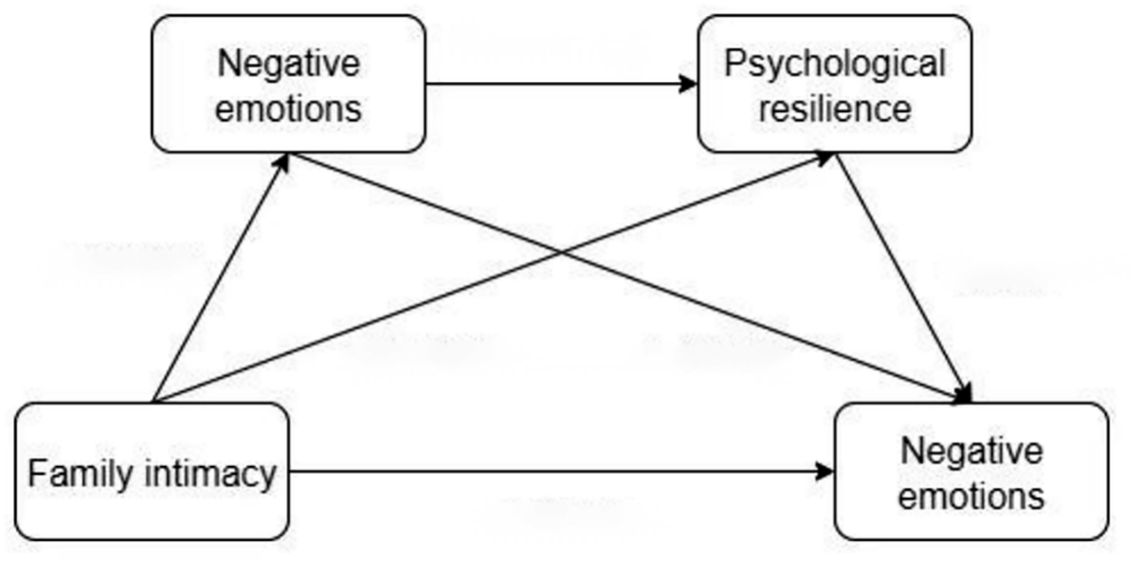
Chain *mediation effects of social support* and psychological resilience.

Collectively, this study proposes four research hypotheses and constructs a chain mediation model, as shown in Figure 1:

*H1*: Family intimacy is negatively associated with college students’ negative emotions.

*H2*: Social support mediates the effect of family intimacy on college students’ negative emotions.

*H3*: Psychological resilience mediates the relationship between family intimacy and college students’ negative emotions.

*H4*: Social support and psychological resilience have a chain mediating effect in the relationship between family intimacy and college students’ negative emotions.

## 3 Methodology

### 3.1 Data sources

After obtaining informed consent from the participants, a questionnaire was distributed to students from 801 universities across 33 provinces (autonomous regions, municipalities, and special administrative regions) in China, excluding Taiwan. A total of 3,589 questionnaires were returned, of which 3,010 were valid (83.87%). The sample included 1,141 males (37.9%) and 1,869 females (62.1%), with ages ranging from 19 to 29 years, and an average age of 20.16 years.

### 3.2 Research tools

The overall questionnaire used in this study consisted of basic demographic information and four specific scales. The demographic section included age, gender (male, female). The four scales assessed the following constructs: Family intimacy, Social Support, Psychological Resilience, Negative Emotions.

#### 3.2.1 Family Environment Scale

This study used the Chinese version of the *Family Environment Scale* (FES-CV), which was developed by Moss et al. and later revised by Fei Lipeng et al. The scale includes 10 dimensions related to family factors. For the purpose of this study, the factor related to intimacy was selected. A dichotomous response format (“Yes-No”) was used, with “Yes” scored as 1 and “No” scored as 0. A higher score indicates greater family intimacy. The Cronbach’s alpha for this scale in the current study was 0.955.

#### 3.2.2 Depression Anxiety Stress Scale-21

The study used the *Depression Anxiety Stress Scale-21* (DASS-21) in its simplified Chinese version, revised by Gong Xu et al., to assess the level of negative emotions in college students. This scale evaluates personal negative emotional experiences or corresponding physiological responses in three dimensions: depression, anxiety, and stress. A Likert four-point scoring system was employed, where higher scores indicate higher levels of negative emotions and lower psychological wellbeing. The Cronbach’s alpha for this scale in the current study was 0.955.

#### 3.2.3 Self-Rating Health Measurement Scale

For measuring social support, this study adopted the *Self-Rating Health Measurement Scale* (SRHMS) developed by Xu et al. (2004). This scale contains 10 dimensions and 3 subscales. For the specific focus of this study, the social support dimension was selected. The scale was developed based on the World Health Organization’s health definition and China’s national conditions, and it effectively reflects individuals’ subjective evaluation and expectations of their social support. The scale uses a 0–10 point scoring system, where higher scores indicate greater social support. The Cronbach’s alpha for this scale in the current study was 0.900.

#### 3.2.4 Connor-Davidson Resilience Scale-10

The *Connor-Davidson Resilience Scale-10* (CD-RISC-10), originally developed by Connor and Davidson and later revised by Zhang et al. (2022), was used in this study to assess psychological resilience. The scale uses a Likert five-point scoring system ranging from 0 (“not at all true”) to 4 (“almost always true”). The total score is the sum of individual item scores, with higher scores indicating stronger psychological resilience. The Cronbach’s alpha for this scale in the current study was 0.949.

## 4 Research results

### 4.1 Common method bias

Since the data collected in this study were based on self-reports from the participants, there may be a potential issue of common method bias. To address this, Harman’s single-factor test was conducted to assess common method bias in the variables of family closeness, negative emotions, social support, and psychological resilience. The results revealed that the first unrotated factor explained only 31.23% of the total variance, which is below the threshold of 40% for common method bias. This indicates that there is no significant issue of common method bias in this study.

### 4.2 Descriptive statistics and correlations

Table 1 summarises means, standard deviations and zero-order correlations for all study variables. Age and gender were weakly associated with the key constructs and were therefore retained as control variables. Family intimacy correlated positively with social support (*r* = 0.19, *p* < 0.001) and psychological resilience (*r* = 0.12, *p* < 0.001), and negatively with negative emotions (*r* = −0.11, *p* < 0.001). Social support and resilience showed moderate, inverse relations with negative emotions (*r* = −0.26 and −0.33, respectively, *p* < 0.001), satisfying the prerequisites for mediation analyses.

**Table 1:** Descriptive statistics and correlation analysis of variables (*n* = 3,010).

Variable	*M*	*SD*	Age	Gender	Family cohesion	Social support	Resilience	Negative emotion
Age	20.16	2.22	1					
Gender	0.38	0.49	0.021	1				
Family intimacy	1.88	0.47	0.008	0.043^*^	1			
Social support	6.91	1.79	0.016	0.009	0.188^**^	1		
Resilience	3.55	0.80	0.028	0.069^**^	0.121^**^	0.408^**^	1	
Negative emotion	0.75	0.61	0.011	0.026	−0.108^**^	−0.261^**^	−0.330^**^	1

*p < 0.05; **p < 0.01; ***p < 0.001; two-tailed test.

### 4.3 Chain-mediation analysis

**Figure 2:**
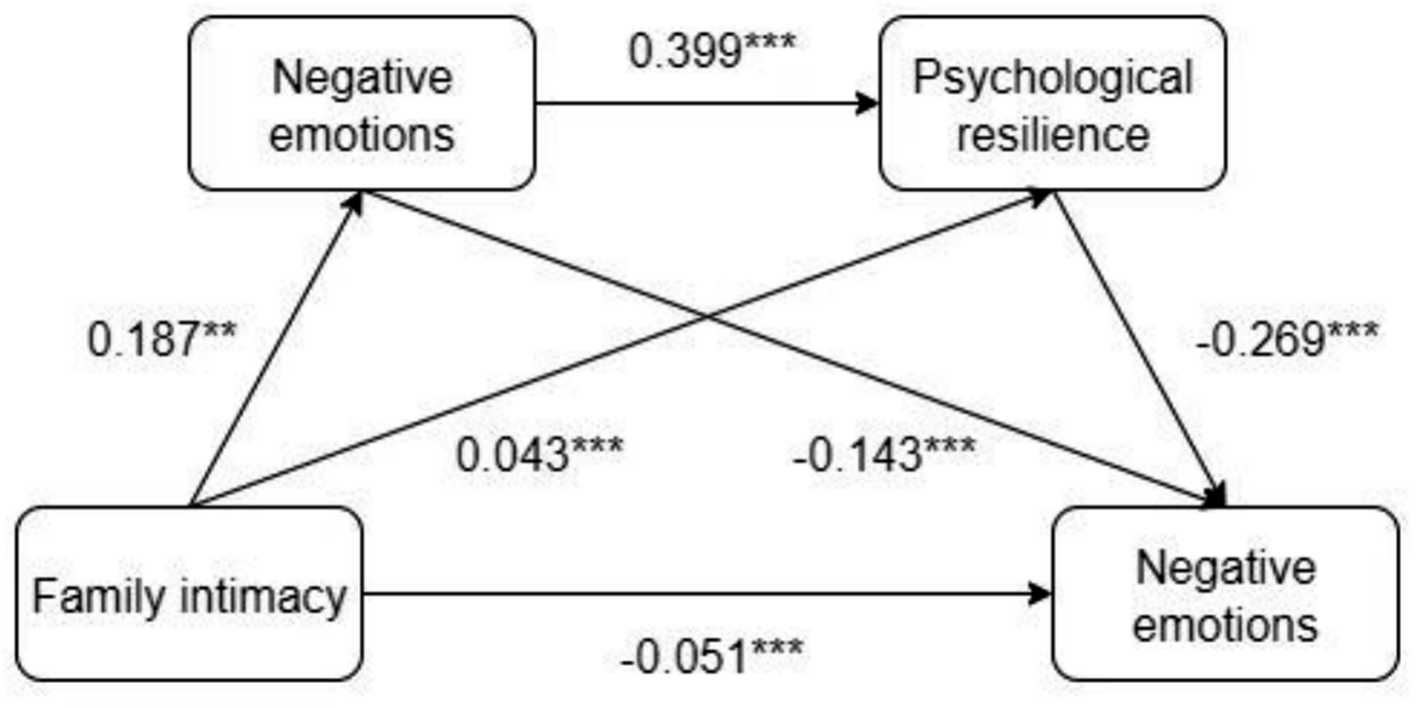
Chain mediation effects of social support and psychological resilience. *p < 0.05, **p < 0.01, ***p < 0.001.

We tested the proposed chain model with PROCESS Model 6 (Hayes, 2022), controlling for age and gender. Figure 2 depicts the path coefficients. Family intimacy significantly predicted higher social support (*β* = 0.187, *p* < 0.001) and greater resilience (*β* = 0.043, *p* < 0.05), while simultaneously predicting lower negative emotions (*β* = −0.051, *p* < 0.01). Social support, in turn, enhanced resilience (*β* = 0.399, *p* < 0.001) and directly reduced negative emotions (*β* = −0.143, *p* < 0.001); resilience exhibited the strongest protective effect (*β* = −0.269, *p* < 0.001). After accounting for these mediators, the direct effect of family intimacy remained significant but attenuated (*β* = −0.066, *p* < 0.01), indicating partial mediation. See Table 2 for details.

**Table 2:** Chain-mediation regression analyses.

Outcome variable	Predictor	*R*	*R^2^*	*F*	*β*	*t*
Social support	Age	0.188	0.035	36.777	0.015	10.454
	Gender				0.001	0.816
	Family Cohesion				0.187	0.034***
Resilience	Age	0.416	0.173	156.903	0.02	2.559
	Gender				0.064	23.602
	Family intimacy				0.043	1.208*
	Social support				0.399	3.824***
Negative emotion	Age	0.365	0.133	92.102	0.021	1.212
	Gender				0.048	2.799
	Family intimacy				−0.051	−2.919**
	Social support				−0.143	−7.585***
	Resilience				−0.269	−14.404***

*p < 0.05, **p < 0.01, ***p < 0.001.

The mediation analysis showed that the total effect of family intimacy on college students’ negative affect was −0.143 (*t* = −6.015, *p* < 0.001, 95% CI [−0.190, −0.097]). Of this, the direct effect accounted for −0.066 (*t* = −2.919, *p* < 0.01, 95% CI [−0.111, −0.022]), corresponding to 46.2% of the total effect. The combined indirect effect was −0.077, explaining the remaining 53.8%. Full details are reported in Table 3.

**Table 3:** Tests of chain-mediation effects.

Effect	Path	Estimate	Boot SE	95% CI	
				LLCI	ULCI
Total indirect effect		−0.077	0.010	−0.097	−0.058
Specific indirect effects	Family intimacy → social support → negative affect	−0.027	0.005	−0.037	−0.018
	Family intimacy → resilience → negative affect	−0.012	0.005	−0.022	−0.002
	Family intimacy → social support → resilience → negative affect	−0.020	0.003	−0.026	−0.015

## 5 Discussion

This study explores the impact of family intimacy on negative emotions among college students and its underlying mechanisms from a family systems perspective, thereby enriching the empirical evidence on the relationship between family intimacy and college students’ mental health. The results indicate a significant negative correlation between family intimacy and negative emotions, suggesting that a warm and supportive family atmosphere can help reduce levels of negative affect in college students. This finding aligns with previous research indicating that the family, as the earliest context for socialization, provides emotional bonds and interaction patterns that serve as crucial psychological resources, exerting a lasting influence on individuals’ emotional experiences and regulatory capacities (Cai et al., 2022; Bowen, 1978; Armsden and Greenberg, 1987). Notably, this direct effect was consistent across gender groups, suggesting that the protective role of family intimacy against negative emotions is stable across genders, a result that echoes findings from cross-cultural studies (Sheeber et al., 2001).

The analysis of the social support pathway further reveals the indirect mechanism through which family intimacy influences college students’ negative emotions. The study found that family intimacy can effectively buffer negative emotions by promoting the establishment and maintenance of social support systems. This result is consistent with Cohen and Wills’ (1985) buffering hypothesis, which posits that social support plays a significant protective role in stress adaptation. Specifically, the family, as a primary source of social support, not only provides direct emotional comfort and material assistance but also shapes individuals’ social skills and resource acquisition abilities through the socialization process, thereby enhancing their capacity to cope with stress (Cutrona, 1996). On the one hand, individuals from highly intimate families are more likely to develop strong communication skills and conflict resolution abilities through daily interactions, laying the foundation for building and maintaining high-quality interpersonal relationships both on and off campus. Empirical studies have shown that a positive family atmosphere can promote the development of adolescents’ social skills and enhance their social integration (Laursen and Collins, 2009). On the other hand, family intimacy strengthens individuals’ sense of belonging and security, increasing their willingness and ability to utilize external social support resources. This process can be explained through attachment theory: the formation of secure attachment makes individuals more inclined to seek help from others in times of difficulty rather than falling into negative self-doubt (Mikulincer and Shaver, 2007). Although the influence of the family may decline with age as peer relationships expand during college years, it continues to play a role by providing emotional security and value identification. This suggests that the effect of family intimacy on social support is not temporary but a long-term factor throughout the socialization process (Kessler et al., 2005).

The analysis of the psychological resilience pathway shows that family intimacy can shape individuals’ resilience traits through emotional interaction and behavioral modeling, thereby influencing mental health. Specifically, frequent and positive emotional expression and problem-solving discussions among family members help individuals form constructive cognitive patterns. Open and sincere emotional exchanges create a safe psychological atmosphere that encourages individuals to express and share their inner experiences. Meanwhile, the process of jointly analyzing problems and exploring solutions enhances individuals’ critical thinking and multi-perspective reasoning abilities. Over time, these positive interaction patterns become internalized as cognitive habits, enabling individuals to maintain emotional stability and proactive coping in the face of challenges (Masten, 2014). Additionally, children learn constructive coping strategies—such as emotion regulation and problem-solving—by observing their parents’ responses to stress. According to Bandura’s (1977) social learning theory, individuals acquire coping strategies by imitating others’ behaviors and attitudes. Parents’ rational and self-regulated behaviors in daily life serve as behavioral models and psychological exemplars for their children. This process not only imparts specific skills but also reinforces a positive attitude toward adversity and a strong sense of self-efficacy. Notably, the sense of security fostered by family intimacy also provides psychological resources that enhance individuals’ confidence and coping abilities when exploring new environments or facing challenges. Research has shown that individuals with high resilience are able to develop effective coping strategies through reflection and experience accumulation after failures or setbacks, thereby continuously improving their self-efficacy and psychological adaptability (Cicchetti, 2010; Wu et al., 2013).

The cross-pathway analysis further reveals a synergistic effect between social support and psychological resilience. Specifically, the social skills enhanced through the social support pathway associated with family intimacy can further promote the development of resilience. Conversely, the proactive coping styles cultivated through the resilience pathway can improve individuals’ efficiency in accessing and utilizing social support resources. This bidirectional reinforcement mechanism is particularly evident in the face of major stressful events, suggesting that the protective effect of family intimacy on mental health is achieved through a complex, multi-level, and multi-pathway system (Masten, 2014; Cohen and Wills, 1985). From a practical perspective, directly improving family economic conditions is often constrained by structural and institutional factors, making it costly and long-term. In contrast, strengthening family emotional bonds through family education guidance and parent–child communication training is more feasible and actionable. Such interventions can optimize family interaction patterns, enhance emotional ties, and improve social support functions. These efforts can not only alleviate college students’ negative emotions in the short term but also promote the long-term development of psychological resilience, laying a solid foundation for coping with future challenges (Chen and Jiang, 2025).

In summary, this study finds that family intimacy can enhance psychological resilience by improving social support, ultimately reducing negative emotions—a chain mediation effect. This indicates that the psychological protective role of family intimacy is not a simple direct effect but a networked influence mechanism involving multiple interrelated mediating variables. As a key context for early socialization, the emotional atmosphere of the family can profoundly and persistently influence how college students cope with difficulties and challenges, providing ongoing support for their mental health.

## Data Availability

The original contributions presented in the study are included in the article/supplementary material, further inquiries can be directed to the corresponding author.
